# Dose-Dependent Effect of Tumor Mutation Burden on Cancer Prognosis Following Immune Checkpoint Blockade: Causal Implications

**DOI:** 10.3389/fimmu.2022.853300

**Published:** 2022-06-03

**Authors:** Ming Zheng

**Affiliations:** ^1^ Institute of Military Cognition and Brain Sciences, Academy of Military Medical Sciences, Beijing, China; ^2^ Beijing Institute of Basic Medical Sciences, Beijing, China

**Keywords:** immune checkpoint blockade (ICB), tumor mutation burden (TMB), hazard ratio (HR), human cancer, overall survival (OS), cytotoxic T-lymphocyte-associated protein 4 (CTLA-4), programmed cell death protein-1 (PD-1), programmed death ligand-1 (PD-L1)

## Introduction

Over the past decade, immunotherapy has revolutionized cancer treatment by inducing durable remission and prolonging patient survival in diverse types of cancer (1). In the landscape of cancer immunotherapies, therapeutically targeting immune inhibitory checkpoints through the blockade of cytotoxic T-lymphocyte-associated protein 4 (CTLA-4), programmed cell death protein-1 (PD-1), and programmed death ligand-1 (PD-L1) are most broadly effective (2, 3). Based on its unprecedented success in multiple types of cancer (4), immune checkpoint blockade (ICB) treatment is now FDA approved for cancer patients with tumor mutation burden (TMB) >10 mutations/megabase (mut/Mb) (5). However, high TMB (>10 mut/Mb) is not associated with improved survival of ICB-treated patients in all types of cancer (6, 7). It is not unexpected that the arbitrary TMB cut-off of 10 mut/Mb fails to predict ICB response, especially considering that TMB levels can widely range from 0.01 mut/Mb to more than 400 mut/Mb (8). Recently, Dr. Yarchoan et al. have reported a positive correlation between TMB and improved overall survival (OS) following ICB treatment in cancer types with higher TMB, but the correlation did not meet statistical significance (9). Hence, although the preliminary finding by Dr. Yarchoan et al. is promisingly positive, there is still a scarcity of data regarding TMB as a continuous prognostic factor that the effect of increasing TMB levels on improved ICB response remains to be elucidated.

## The Analytical Framework Based on Non-Linear Model OF Hazard Ratio (HR) Across The Continuous Spectrum OF Biomarker

Previously, we have developed an analytical framework for modeling the non-linear relationship of prognostic data with the continuous spectrum of biomarker (10). This analytical framework introduced flexibility into Cox proportional hazards regression model based on the spline-based smoothing method (11, 12). A statistical approach described as smoothHR (13) was applied to construct the natural cubic regression spline curve of hazard ratio (HR) with 95% confidence intervals (CIs) (14). To the best of our knowledge, this method allowed us to investigate the non-linear relationship between TMB gradient and ICB response for the first time (**Supplementary Figure 1**).

The non-linear HR analysis was conducted using the Morris’s cohort of 1,662 patients with cancer, who had received ICB treatment through the blockades of PD1, PD-L1, and CTLA4 (**Supplementary Figure 2**). The patients’ TMB values were measured by next-generation sequencing (NGS) using the MSK-IMPACT panel previously, and the detailed clinical information, including cancer types, sex, age, ICI drug class, year of ICB start, and OS, were retained from Morris’s study (15). Here, we presented the data modeling the TMB gradient as a continuous risk predictor of the OS following ICB treatment. The HRs were calculated by univariate and multivariate analyses of Cox proportional hazard regression taking TMB = 0 mut/Mb as a reference, and the smoothing estimate of HR was calculated across the spectrum of TMB values. Multivariate analysis was conducted using covariates of cancer types, sex, age, ICI drug class, and year of ICB start. In order to rule out the outliers at the distal end of the log-HR curve, truncation was performed to remove TMB values above the 95th percentile. Next, the log-HR curve with 95% CIs was plotted with a straightforward interpretation that the 95% CIs above or below 0 is equivalent to a significance of two-sided *p* < 0.05.

## The Dose-Dependent Effect of Tumor Mutation Burden (TMB) on the Cancer Prognosis Following Immune Checkpoint Blockade (ICB) Treatment

**Figure 1:**
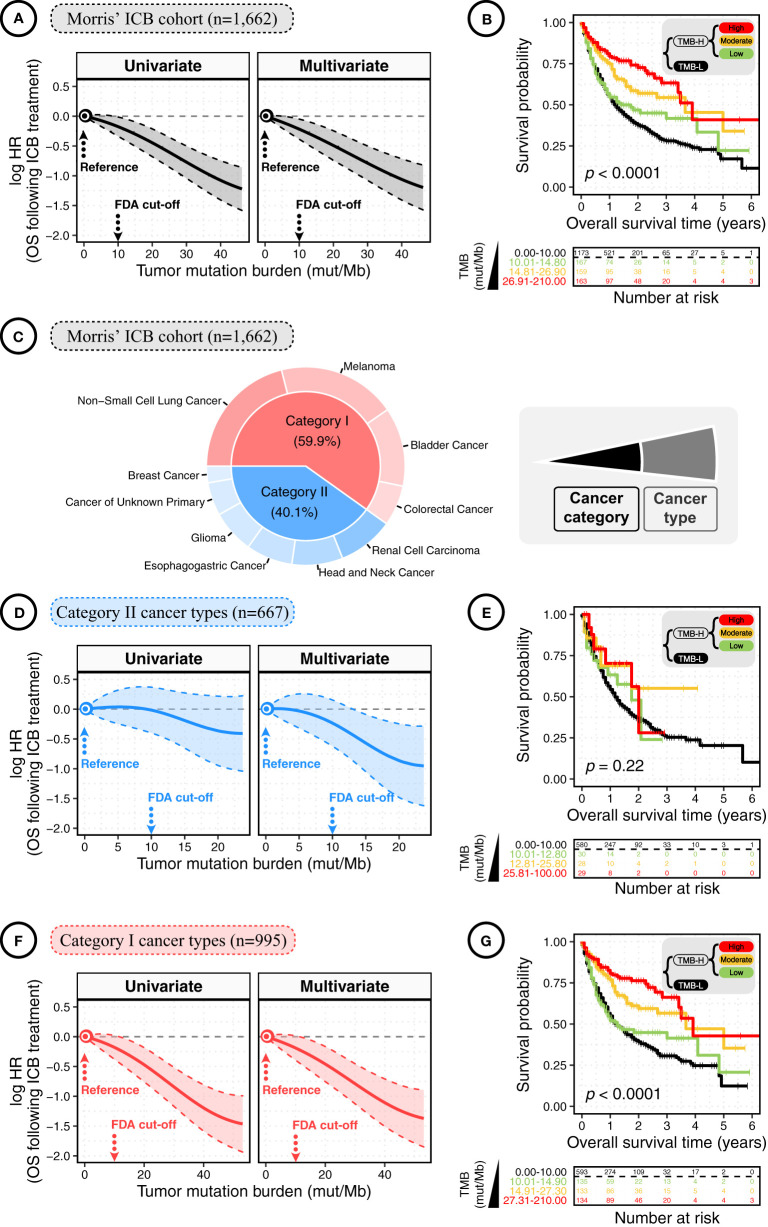
The dose-dependent association between tumor mutation burden (TMB) and overall survival (OS) in cancer patients following immune checkpoint blockade (ICB) treatment. **(A)** The smoothing estimate of hazard ratios (HRs) with 95% confidence intervals (CIs) across the spectrum of TMB gradient. The continuous log-HR curves (solid line) with 95% CIs (shading) show the association of TMB with the OS following ICB treatment in total patients from the Morris’ ICB cohort. The HRs were fitted by univariate and multivariate Cox proportional hazards regressions. Multivariate analysis was conducted using covariates of cancer type, sex, age, ICI drug class, and year of ICB start. To rule out the outliers at the distal end of log-HR curve, truncation was performed to remove TMB values above the 95th percentile. The x-axis shows TMB values (mut/Mb), and the y-axis shows HR, taking the patients with TMB = 0 mut/Mb as the reference. **(B)** The Kaplan-Meier curves show the OS of total cancer patients following ICB treatment according to TMB. High-TMB (TMB-H) and low-TMB (TMB-L) Patients were defined using the FDA-approved TMB cutoff of 10 mut/Mb; next, TMB-H patients were divided equally into low, moderate, and high TMB-H subgroups. The significance was measured by the log-rank test. The bottom panel shows the number of patients at risk every one year. **(C)** The pie plot shows the percentage of patients by cancer type and category. Category I cancers were cancer types where CD8^+^ TIL-T-cell levels positively correlated with neoantigen loads, while no such correlation was observed in category II cancers. Category I cancers: non-small cell lung cancer, melanoma, bladder cancer, and colorectal cancer; category II cancers: renal cell carcinoma, head and neck cancer, oesophagogastric cancer, glioma, cancer of unknown primary, and breast cancer. **(D)** The continuous log-HR curves (solid line) with 95% CIs (shading) show the association of TMB with the OS following ICB treatment in cancer patients of category II cancer types. **(E)** The Kaplan-Meier curves show the OS following ICB treatment according to TMB in cancer patients of category II cancer types. The bottom panel shows the number of patients at risk every one year. The significance was measured by the log-rank test. **(F)** The continuous log-HR curves (solid line) with 95% CIs (shading) show the association of TMB with the OS following ICB treatment in cancer patients of category I cancer types. **(G)** The Kaplan-Meier curves show the OS following ICB treatment according to TMB in cancer patients of category I cancer types. The significance was measured by the log-rank test.

In both univariate and multivariate analyses of total patients, the smoothing log-HR curves showed that TMB was significantly associated with an improved OS following ICB treatment in a dose-dependent manner (**Figure 1A**). The log-HR curves revealed similar trends in univariate and multivariate analyses, indicating TMB as an independent prognostic factor of ICB response. In agreement with the above findings, Kaplan-Meier analyses showed a significant difference in OS between patients with high-TMB (TMB-H) versus low-TMB (TMB-L) tumors (*p* < 0.0001; **Figure 1B**). TMB-H and TMB-L patients were defined using the FDA-approved TMB cutoff — 10 mut/Mb; and, TMB-H patients were further divided equally into low, moderate, and high TMB-H subgroups. For each decrease in TMB levels, there was a stepwise reduction in OS. The TMB-L patients had the poorest survival with a median OS time of 1.25 years (95% CIs: 1.08 to 1.42). By contrast, the low, moderate, and high TMB-H subgroups had median OS times of 1.42 years (95% CIs; 0.92 to NR [not reached]), 3.67 years (95% CIs: 2.00 to NR), and 3.92 years (95% CIs: 3.42 to NR), respectively.

## The Benefit of ICB Treatment Is Increased With TMB level but Also Depends on the Context of Cancer Types

A previous study has reported that the assessment of ICB response is related to the context of cancer categories defined by whether CD8 T-cell infiltration is positively correlated with neoantigen load (7). As previously reported, we divided the total patients into category I and II cancers (**Figure 1C**). Category I cancers include non-small cell lung cancer, melanoma, bladder cancer, and colorectal cancer, where the levels of CD8^+^ tumor-infiltrating T cells were positively correlated with the neoantigen loads of tumor, while no such correlation was observed in the rest cancers of category II (7). As shown in **Figure 1D**, the log-HR curves for category II cancers were generally constant and slightly dropped at the distant end where the TMB level was above 15 mut/Mb, and the range of 95% CIs of HR was relatively broad, showing a generally insignificant association. Consistently, the Kaplan-Meier plot showed no significant difference in OS between TMB-L and TMB-H patients with category II cancers (**Figure 1E**).

Comparatively, in category I cancers, HRs significantly decreased with increasing TMB values (**Figure 1F**). A consistent finding was observed in the Kaplan-Meier analyses. As shown in **Figure 1G**, the TMB-L patients with category I cancers had the poorest survival with median OS times of 1.25 years (95% CIs: 1.08 to 1.50), and the low, moderate, and high TMB-H subgroups had median OS times of 1.25 years (95% CIs: 0.83 to NR), 3.67 years (95% CIs: 2.67 to NR), and 3.92 years (95% CIs: 3.41 to NR), respectively. Thus, the benefit of ICB treatment is increased with elevated TMB levels, but it mainly depends on the context of cancer types.

## Discussion

ICB treatment was initially demonstrated efficacy and FDA approved for patients with melanoma, and later expanded to other types of cancer (16). The 2020 FDA approval of pembrolizumab (anti-PD-1) in TMB-H patients represents a transformative event of ICB therapies (5). Since then, ICB therapies have been extended to a remarkably skyrocketing number of patients in a broad range of cancer types. However, as we look to the foreseeably inspiring future of ICB-based immunotherapy, many aspects of clinical practice and biological mechanisms underlying ICB remain to be elucidated. At present, one of the major factors affecting ICB response is the absolute level of TMB (4). To the best of our knowledge, this is the first study showing that the increasing level of TMB has a significant effect on the improved prognosis following ICB treatment in a dose-dependent manner. The dose-dependent relationship strongly implies the causal role of TMB in ICB response, with important clinical and therapeutic implications for ICB-based immunotherapy.

In this study, we found that TMB-H tumors were substantially more responsive to ICB treatment. ICB treatment inhibits tumor progression by boosting the anti-tumor immunity of cytotoxic T cells (CTLs) (17). Based on blocking the co-inhibitory pathways, anti-CTLA-4 enhances the co-stimulation signaling and lowers the activation threshold of T cell receptor (TCR) (18, 19), while anti-PD-1/PD-L1 reduces the requirement for TCR signaling and reinvigorates exhausted CTLs (20). As indicated by the above-stated mechanisms, ICB treatment works better in tumors recognizable by CTLs. T-cell recognition of tumor cells is based on the interaction of TCRs with major histocompatibility complex (MHC)-presented neoantigens (21, 22). Indeed, neoantigens are mutated proteins derived from tumor somatic mutations. Before this study, TMB has been typically considered as a logical surrogate of neoantigen load. Therefore, it seems reasonable to assume that TMB-H tumors, harboring a greater immunogenicity of higher abundance and variety of neoantigens, are statistically more likely to be recognized and killed by CTLs.

Previous studies have exhaustively tested the robustness of TMB-H as a predictive biomarker for improved ICB response (6, 7). Consistent with previous studies, the finding in this study also suggests that the higher the TMB level, the better the ICB response. However, these exciting results were observed only in category I cancers, such as non-small cell lung cancer, melanoma, bladder cancer, and colorectal cancer. These cancers are much more likely to be exposed to mutagens such as chemical carcinogens and ultraviolet radiation that induce somatic mutations. Actually, category I cancers have relatively higher TMB levels (6), where immunotherapy was initially discovered to be successful (23).

Unlike category I cancers, most category II cancers have relatively lower TMB levels (6), poorer inherently immunogenicity, and less T cell infiltration (7). In category II cancers, such as breast cancer and glioma, TMB-H was not associated with a favorable prognosis but even related to an unfavorable ICB response (6). Moreover, according to this study, the dose-dependent relationship between TMB and ICB response is most significant in category I cancers but generally insignificant in category II cancers. In conclusion, the favorable ICB responsiveness is increased with increasing TMB levels, but also depends on the context of cancer types. Consequently, we should be cautious about generalizing the present success of ICB treatment to other cancer types directly. Future studies should focus on the cancer type specificity of ICB response in patients with TMB-H tumors.

## Author Contributions

MZ conceived the project, developed the method, conducted data analysis, and wrote the manuscript. MZ supervised this project and is responsible for the overall content.

## Funding

This project was supported by the National Natural Science Foundation of China (32100739) to MZ.

## Conflict of Interest

The author declares that the research was conducted in the absence of any commercial or financial relationships that could be construed as a potential conflict of interest.

## Publisher’s Note

All claims expressed in this article are solely those of the authors and do not necessarily represent those of their affiliated organizations, or those of the publisher, the editors and the reviewers. Any product that may be evaluated in this article, or claim that may be made by its manufacturer, is not guaranteed or endorsed by the publisher.

## Supplementary Material

Supplementary Figure 1 The analytical framework is based on non-linear model of hazard ratio (HR) across the continuous spectrum of biomarker. Based on Cox proportional hazard regression, the pointwise hazard ratio (HR) was calculated for each value of tumor mutation burden (TMB; mut/Mb), taking TMB = 0 mut/Mb as the reference. The association between TMB and patient survival was assessed by both univariate and multivariate Cox regression.

Supplementary Figure 2
**(A)** The pie plot shows the percentage of patients by ICI drug class and cancer type. ICI drug class: anti-PD-1 or PD-L1; anti-CTLA-4; and a combination of anti-CTLA-4 and anti-PD-1/PD-L1 therapies. Category I cancers were cancer types where CD8+ TIL-T cell levels positively correlated with neoantigen loads, while no such correlation was observed in category II cancers. Category I cancers: non-small cell lung cancer, melanoma, bladder cancer, and colorectal cancer; category II cancers: renal cell carcinoma, head and neck cancer, oesophagogastric cancer, glioma, cancer of unknown primary, and breast cancer.
