# Supplementary material for: Dose-Dependent Effect of Tumor Mutation Burden on Cancer Prognosis Following Immune Checkpoint Blockade: Causal Implications
Source: Front Immunol. 2022 Jun 3;13:853300. doi: 10.3389/fimmu.2022.853300 (PMC9203856; doi:10.3389/fimmu.2022.853300)
Supplement: Supplementary Figure 1 — The analytical framework is based on non-linear model of hazard ratio (HR) across the continuous spectrum of biomarker. Based on Cox proportional hazard regression, the pointwise hazard ratio (HR) was calculated for each value of tumor mutation burden (TMB; mut/Mb), taking TMB = 0 mut/Mb as the reference. The association between TMB and patient survival was assessed by both univariate and multivariate Cox regression. [file DataSheet_1.pdf]

Supplementary Material

Dose-dependent effect of tumor mutation burden on cancer prognosis following immune checkpoint blockade: causal implications

Ming Zheng[1,2,*]

Authors' Affiliations:

1. Institute of Military Cognition and Brain Sciences, Academy of Military Medical Sciences, 27 Taiping Road, Beijing 100850, China
2. Beijing Institute of Basic Medical Sciences, 27 Taiping Road, Beijing 100850, China

* Corresponding author.

Correspondence to: **Ming Zheng, MD, Ph.D.**, Institute of Military Cognition and Brain Sciences, Academy of Military Medical Sciences, 27 Taiping Road, Beijing 100850, China.

E-mail: mmzheng@fmmu.edu.cn or zhengming_china@163.com;

ORCID ID: https://orcid.org/0000-0002-3651-7701;

Researcher ID: https://publons.com/researcher/4413823/ming-zheng.

This PDF file includes:

Abstract

Supplementary Figure

Abstract

In 2020, the US Food and Drug Administration (FDA) approved the treatment of immune checkpoint blockade (ICB) in cancer patients with high tumor mutation burden (TMB). Since then, ICB treatment has been extended to a remarkably skyrocketing number of patients in a broad range of cancer types. However, despite the foreseeably inspiring future for ICB-based immunotherapy, there is still a scarcity of data regarding TMB as a continuous prognostic factor that the effect of increasing TMB levels on improved ICB response remains to be elucidated. To the best of our knowledge, this is the first study to demonstrate the dose-dependent relationship of TMB with the improved overall survival (OS) following ICB treatment. This finding strongly implies the causal role of TMB in ICB response, with important clinical and therapeutic implications for ICB-based immunotherapy.

Supplementary Figure 1

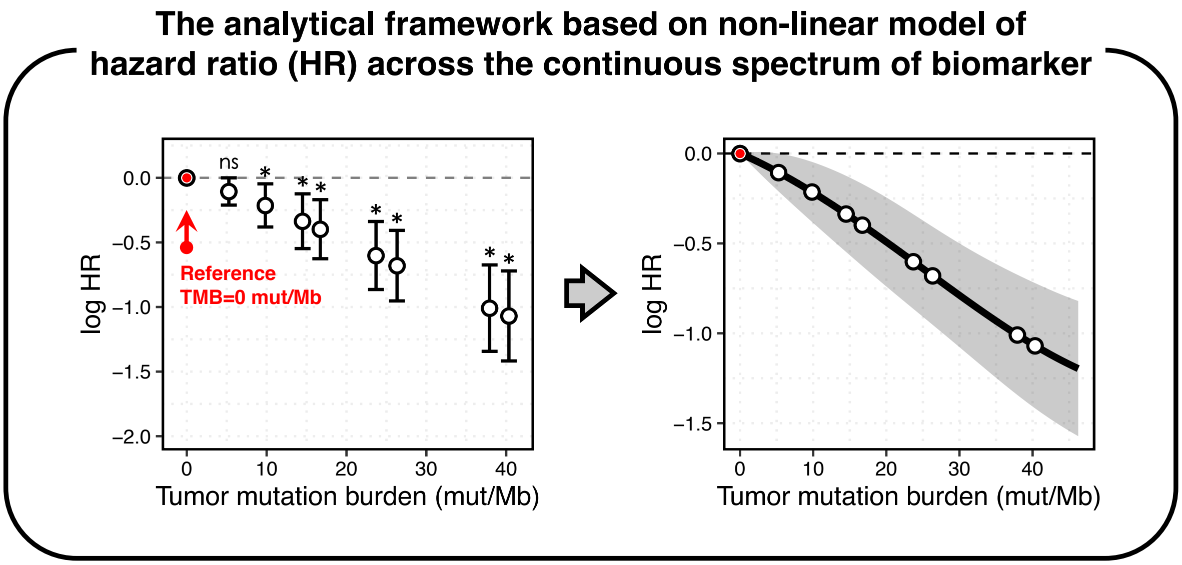

Supplementary Figure 1. The analytical framework is based on non-linear model of hazard ratio (HR) across the continuous spectrum of biomarker. Based on Cox proportional hazard regression, the pointwise hazard ratio (HR) was calculated for each value of tumor mutation burden (TMB; mut/Mb), taking TMB = 0 mut/Mb as the reference. The association between TMB and patient survival was assessed by both univariate and multivariate Cox regression.

Supplementary Figure 2

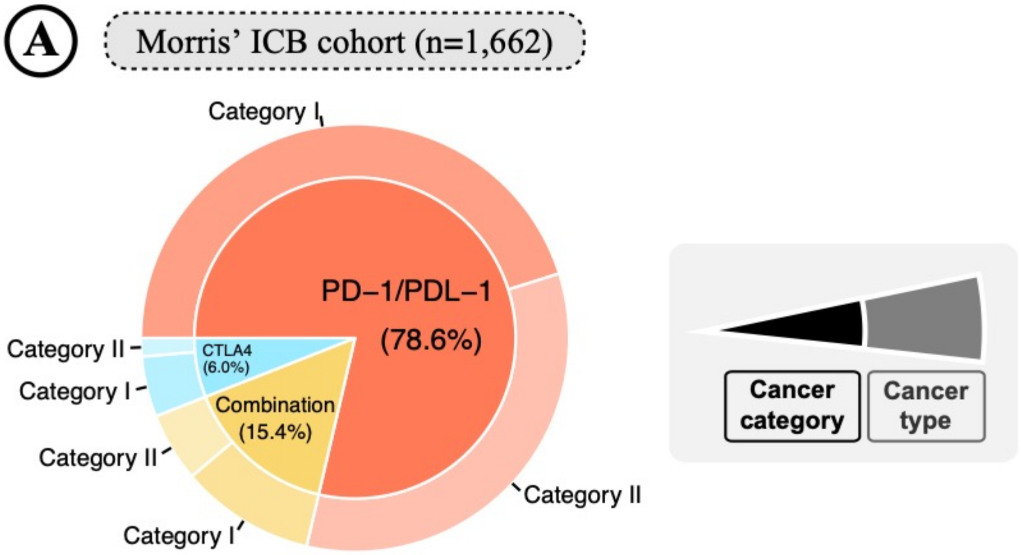

Supplementary Figure 2. (A) The pie plot shows the percentage of patients by ICI drug class and cancer type. ICI drug class: anti-PD-1 or PD-L1; anti-CTLA-4; and a combination of anti-CTLA-4 and anti-PD-1/PD-L1 therapies. Category I cancers were cancer types where $CD8^{+}$ TIL-T-cell levels positively correlated with neoantigen loads, while no such correlation was observed in category II cancers. Category I cancers: non-small cell lung cancer, melanoma, bladder cancer, and colorectal cancer; category II cancers: renal cell carcinoma, head and neck cancer, oesophagogastric cancer, glioma, cancer of unknown primary, and breast cancer.
